# Effects of Selective Retina Therapy on Central Serous Chorioretinopathy with Serous Pigment Epithelial Detachments

**DOI:** 10.3390/jcm15103905

**Published:** 2026-05-19

**Authors:** Dayeong Kim, Seung Hee Jeon, Young-Jung Roh

**Affiliations:** 1 Graduate School, College of Medicine, The Catholic University of Korea, Seoul 06591, Republic of Korea; airadin2000@naver.com; 2 Department of Ophthalmology and Visual Science, Yeouido St. Mary’s Hospital, College of Medicine, The Catholic University of Korea, Seoul 07345, Republic of Korea; jsh881107@catholic.ac.kr

**Keywords:** central serous chorioretinopathy, pigment epithelial detachment, retinal pigment epithelium, selective retina therapy, subretinal fluid

## Abstract

**Background/Objective:** This study’s aim is to evaluate the anatomical and functional effects of selective retina therapy (SRT) in patients with central serous chorioretinopathy (CSC) accompanied by serous pigment epithelial detachment (PED). **Methods:** This retrospective study included 32 eyes from 32 patients with CSC and serous PED treated with SRT. Pulse energy and micropulse number were adjusted based on test spot visibility on fundus photographs. Best-corrected visual acuity (BCVA; logMAR), central foveal thickness (CFT), subretinal fluid (SRF) height, PED height, and subfoveal choroidal thickness were assessed at baseline and at 1, 2, and 3 months post-treatment. Retinal sensitivity was evaluated using microperimetry at baseline and 3 months. **Results:** At 3 months after SRT, complete SRF resolution was achieved in 78.1% of eyes (25/32). Mean BCVA improved significantly from 0.29 ± 0.30 logMAR at baseline to 0.20 ± 0.29 logMAR (*p* = 0.006). Mean CFT decreased from 284.7 ± 91.3 µm to 165.7 ± 94.8 µm (*p* < 0.001). Mean SRF height decreased from 150.5 ± 74.6 µm to 20.9 ± 48.3 µm (*p* < 0.001), and mean PED height decreased from 101.7 ± 96.9 µm to 33.3 ± 37.6 µm (*p* < 0.001). Retinal sensitivity showed a non-significant improvement at 3 months (*p* = 0.108). Reduction in PED height was moderately correlated with reduction in SRF height (r = 0.446, *p* = 0.011). **Conclusions:** SRT was associated with reductions in PED and SRF in CSC. These findings should be interpreted cautiously given the absence of a control group and the potential for spontaneous changes in SRF and PED.

## 1. Introduction

Central serous chorioretinopathy (CSC) is a retinal disease characterized by idiopathic serous detachment of the neurosensory retina that can occur with or without pigment epithelial detachment (PED) [1]. Its annual incidence rate is 9.9 per 100,000 for men and 1.7 per 100,000 for women [2,3]. The underlying pathophysiological mechanism of CSC is believed to involve choroidal vascular hyperpermeability, which triggers PED development and retinal pigment epithelium (RPE) dysfunction, ultimately resulting in subretinal fluid (SRF) accumulation [2]. While acute CSC tends to show good visual recovery within 1–4 months without treatment, chronic or recurrent CSC usually results in permanent visual impairment due to persistent SRF [4,5,6]. Therefore, eliminating SRF is crucial when treating patients with CSC since its complete resolution promotes visual recovery or prevents further vision loss.

PED is defined as the separation between the RPE and underlying Bruch’s membrane [7]. Previous studies have reported that PEDs are visible on optical coherence tomography (OCT) images in 56–96% of CSC-affected eyes [8,9,10]. These PEDs may or may not be associated with SRF, and their development appears to be associated with underlying choroidal dysfunction, including dilation and increased numbers of choroidal vessels [11]. Since persistent PEDs are associated with poor visual outcomes [5,6], facilitating resolution of PEDs may be clinically relevant in the management of CSC.

Although no standardized treatment currently exists for CSC, various treatments, including conventional laser photocoagulation, photodynamic therapy (PDT), intravitreal injection of vascular endothelial growth factor (VEGF) inhibitors, subthreshold micropulse laser (SML), and selective retina therapy (SRT), have been reported [11]. Because conventional laser photocoagulation can cause adverse effects such as central scotoma and choroidal neovascularization [12,13], subthreshold laser modalities, including SML and SRT, have been developed to mitigate these limitations. While SML stimulates the RPE to secrete heat shock proteins, without causing any tissue damage, SRT induces selective RPE damage and subsequent RPE proliferation, resulting in the formation of a new RPE layer [14].

To achieve adequate selective RPE damage, two endpoints have been used to titrate energy during SRT. These include the absence or presence of visible spots on fundoscopy and fundus fluorescein angiography (FFA), respectively, because laser energy absorption varies with individual differences in retinal pigmentation. SRT is currently the only therapeutic modality approved by the Korean Ministry of Food and Drug Safety for treating CSC.

Compared to other “non-destructive” subthreshold laser treatments, SRT directly targets the RPE and induces RPE proliferation and migration. This mechanism suggests a strong potential to influence the morphology of PEDs, which are mainly composed of RPE cells. However, published evidence regarding the effects of SRT on PEDs in CSC remains limited. Therefore, this study aimed to assess the effects of SRT on PEDs and related anatomical and functional outcomes in patients with CSC.

## 2. Materials and Methods

The medical charts of 190 consecutive patients (195 eyes) who underwent SRT for CSC between December 2023 and April 2025 were reviewed in this retrospective study. All patients had previously provided written informed consent for selective retina therapy after being informed of the potential risks, including retinal burns and central scotomas. This retrospective study protocol adhered to the tenets of the Declaration of Helsinki and was approved by the Institutional Review Board of Yeouido St. Mary’s Hospital of the Catholic University of Korea (SC25RISI0066), which waived the requirement for additional informed consent for this chart review.

The present study did not aim to evaluate all patients with CSC treated with SRT, but rather focused on a specific subgroup of patients with CSC in whom the angiographic leakage site was anatomically associated with a serous PED, while excluding lesions with features suggestive of choroidal neovascularization. This design was intended to allow a focused assessment of PED-specific morphological changes following SRT.

Eyes with CSC that met predefined inclusion and exclusion criteria were included (Table 1). Eligible eyes were required to demonstrate visual symptoms lasting for at least 3 months, fovea-involving subretinal fluid on OCT, and a serous PED showing a homogeneous hyporeflective signal on OCT. In addition, angiographic leakage was required to originate primarily from the serous PED on fluorescein and/or indocyanine green angiography (ICGA), and a minimum of 3 months of post-SRT follow-up was required for inclusion.

Eyes were excluded if they had other macular diseases, including non-CSC entities such as age-related macular degeneration, polypoidal choroidal vasculopathy, or pathological myopia. Any evidence of choroidal neovascularization detected on multimodal imaging, including OCT, FFA, ICGA, or OCT angiography, was grounds for exclusion. Additional exclusion criteria included fibrous or vascular PEDs showing heterogeneous hyperreflective signals suggestive of neovascular tissue on OCT, serous PEDs with angiographic leakage not primarily originating from the PED, a history of conventional macular laser photocoagulation or PDT, intravitreal anti-VEGF injection within 3 months before SRT, use of local or systemic steroids, acetazolamide, or aldosterone antagonists within 12 months before SRT, and intraocular surgery within 12 months before SRT. This selective inclusion strategy was adopted to reduce phenotypic heterogeneity and to facilitate a more reliable evaluation of serous PED-associated anatomical and functional responses to SRT.

A single physician (YJR) performed SRT using a Q-switched Nd:YLF 527 nm laser device (Macufocus; Threshold Inc, Seoul, Republic of Korea), with a 200 µm spot-size diameter, 1.7 µs single micropulse duration, and a 100 Hz pulse-repetition rate. This device was approved for CSC treatment by the Korean Ministry of Food and Drug Safety. All SRT procedures were performed under pharmacological pupil dilation. A retinal contact lens (Ocular Mainster Focal/Grid; Ocular Instruments, Bellevue, WA, USA) was used. Although well-titrated SRT spots are invisible during laser irradiation, faintly visible SRT spots can be observed on color fundus photography (CFP) approximately 1 h post-SRT. Using the delayed fundus visibility of SRT spots, SRT was performed by adjusting the micropulse number and pulse energy according to a previously described fundus-image-guided strategy [15,16,17].

**Figure 1:**
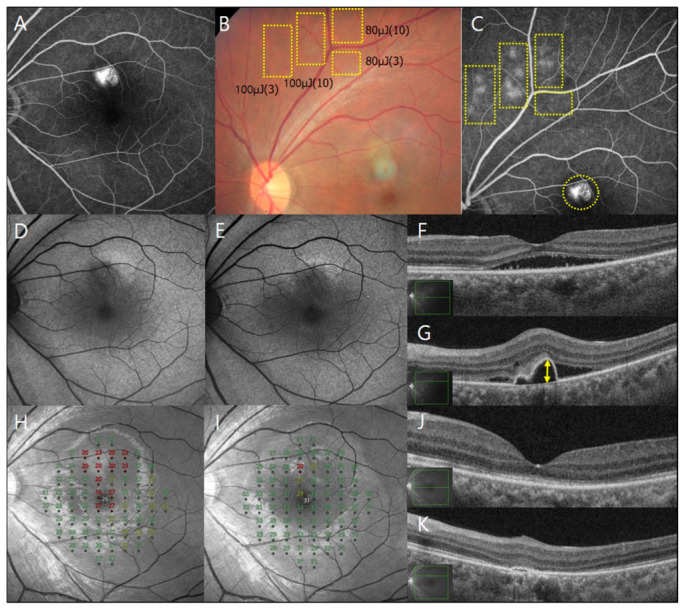
Case 1. A representative patient with a 3-month history of blurred vision in the left eye. (**A**) Late-phase fundus fluorescein angiography (FFA) shows diffuse hyperfluorescence in the juxtafoveal region. (**B**) Color fundus photography obtained 1 h after test spot irradiation demonstrated multiple test spot pairs within the yellow rectangular boxes. Among them, all five pairs produced using 100 μJ and 10 micropulses were identifiable, whereas only four corresponding test spots produced using 100 μJ and three micropulses were visible. At 80 μJ, fewer test spots were detectable. (**C**) FFA performed 1 h after test spot irradiation demonstrated weaker angiographic leakage with three micropulses than with 10 micropulses at the same pulse energy. Based on the titration protocol, the treatment energy was determined as 100 μJ using a bundle of three micropulses when 100 μJ with 10 micropulses was barely visible. Treatment spots of 100 μJ with three micropulses were applied at pigment epithelial detachment (PED)-related leaks (yellow circle). No changes were observed in fundus autofluorescence from baseline (**D**) to 3 months post-treatment (**E**). (**F**) Optical coherence tomography (OCT) conducted at baseline shows subretinal fluid (SRF) in the fovea and serous PED at the leakage area in the juxtafoveal region. (**G**) The PED height (yellow arrow) was measured at the highest PED point. The mean deviation of retinal sensitivity improved from −2.58 dB at baseline (**H**) to +1.03 dB at 3 months. Numbers indicate retinal sensitivity values (dB). Red and dark-colored areas in the Compass microperimetry maps indicate relatively decreased retinal sensitivity (**I**). The OCT showed the complete resolution of SRF in the fovea (**J**) and PED in the juxtafoveal region (**K**).

Briefly, multiple pairs of preliminary test spots were created with 20 µJ increments of laser pulse energy and a bundle of micropulses (three or 10) around the major arcade vessels. SRT test spots classified as barely visible were observed on CFP approximately 1 h post-irradiation. According to the titration protocol, the treatment pulse energy was determined as the lowest pulse energy that produced barely visible test spots when delivered as a bundle of 10 micropulses. The same energy, delivered as a bundle of three micropulses, was applied to PED-associated leaks at one-spot spacing (Figure 1). Since treatment targeted PED-associated leaks, the SRT-treated area encompassed the PED.

### 2.1. Clinical Measures

All patients underwent slit-lamp examination and assessment of best-corrected visual acuity (BCVA), expressed as the logarithm of the minimum angle of resolution (logMAR), at baseline and at 1, 2, and 3 months post-SRT. Multimodal imaging was performed, including CFP (CF-60UVi; Canon Inc., Ota, Japan), FFA, ICGA, fundus autofluorescence (FAF) (Heidelberg Retina Angiograph 2; Heidelberg Engineering, Heidelberg, Germany), and swept-source OCT (DRI OCT Triton, Topcon, Tokyo, Japan). A 7 × 7 mm volume OCT scan centered on the fovea was used to determine SRF presence and measure the central foveal thickness (CFT), subfoveal choroidal thickness (SFCT), SRF height (defined as the maximum distance between the outer border of the neurosensory retina and the RPE at the foveal center), and PED height (defined as the maximum distance between Bruch’s membrane and the RPE within the detachment).

PED height was measured only in PEDs anatomically associated with the angiographic leakage targeted by SRT. When multiple leakage-associated PEDs were present, the PED with the greatest height was selected to improve measurement reproducibility and to provide a consistent representative anatomical parameter across patients. PEDs without angiographic leakage were excluded because they were not direct treatment targets.

The CFT, SRF height, PED height, and SFCT were measured using the caliper tool in the IMAGENET 6.0 software (Topcon). If multiple PEDs were observed in a patient, the PED with the highest height was selected for measurement (Figure 1). All measurements were performed according to a predefined protocol. The three retina specialist co-authors conducted the image assessments. For each case, two specialists independently evaluated qualitative OCT features, including hyperreflective signals within PEDs and the association between leakage sites and PEDs. Discrepancies in qualitative assessments were resolved by consensus with a third specialist.

CFP, FAF, and OCT images were obtained at baseline and at 1, 2, and 3 months post-SRT. FFA and ICGA were performed to confirm leakage points. On the treatment day, CFP and FFA were performed 1 h after test spot irradiation to determine the pulse energy and number of micropulses required to generate treatment spots. Additional SRT was performed at 2 months using the same laser parameters as in the initial treatment if SRF persisted in the fovea. Outcomes at 3 months reflect the predefined re-treatment strategy.

Retinal sensitivity was measured using automated compass microperimetry (CMP) (Centervue, Padova, Italy) with active retinal tracking. Mean deviation (MD) was calculated automatically using microperimetry. A previous report deemed the test–retest reliability of microperimetry to be appropriate, and the test was similarly regarded as reliable in the present study when the false-positive rate was ≤18% [18]. Microperimetry was conducted at baseline and at 3 months post-treatment. Given that previously reported pointwise test–retest variability in microperimetry generally ranges from approximately 4 to 7 dB across retinal diseases [19], a more conservative threshold of ≥8 dB reduction in pointwise sensitivity, limited to the SRT-treated area, was applied to identify potentially treatment-related functional changes.

### 2.2. Statistical Analysis

Changes in the mean BCVA, CFT, SFCT, SRF height, and PED height from baseline to 1, 2, and 3 months post-SRT were analyzed with paired *t*-tests. The change in the mean MD of retinal sensitivity from baseline to 3 months was analyzed using a paired *t*-test. Pearson’s correlation coefficient was calculated to assess the association between PED and SRF heights. Comparisons between eyes with and without prior anti-VEGF treatment were performed using the Mann–Whitney U test for continuous variables and Fisher’s exact test for categorical variables. Statistical significance was defined as *p* < 0.05. All analyses were performed by the first author (D.K.) using IBM SPSS Statistics for Windows, version 24.0 (IBM Corp., Armonk, NY, USA).

## 3. Results

**Figure 2:**
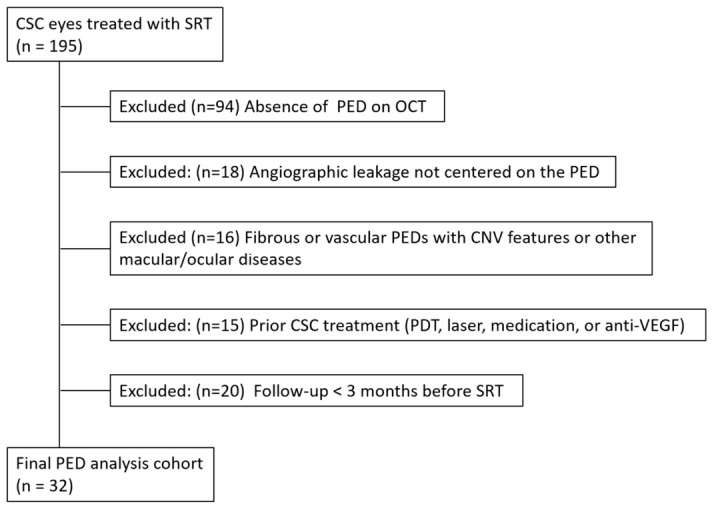
Selection flow chart of study cohort.

A total of 32 eyes from 32 patients with chronic CSC accompanied by serous PEDs were analyzed according to the inclusion and exclusion criteria (Figure 2). Of these 32 eyes, nine had also been included in a separate prospective cohort study [15]. However, the present analysis was conducted to assess serous PED-associated leakage and related anatomical and functional outcomes following SRT. Sensitivity analyses excluding the nine overlapping eyes demonstrated similar statistically significant reductions in SRF and PED heights at 3 months (Supplementary Table S1).

The outcomes at 3 months reflect the predefined re-treatment protocol applied when SRF persisted after initial SRT. Most patients (78.1%) were male, with a mean age of 45.9 ± 10.9 years. The mean duration of the current symptoms was 9.3 ± 11.3 months. Regarding the shape of PEDs, 84.4% (27/32) were dome-shaped, and 15.6% (5/32) were flat (Table 2).

A total of 195 eyes with central serous chorioretinopathy (CSC) treated with selective retina therapy (SRT) were initially screened. Eyes were excluded due to absence of pigment epithelial detachment (PED) on optical coherence tomography (OCT), angiographic leakage not primarily originating from the PED, fibrous or vascular PEDs with features suggestive of choroidal neovascularization (CNV) on multimodal imaging, a history of prior CSC treatment including photodynamic therapy (PDT), laser photocoagulation, or recent intravitreal anti-vascular endothelial growth factor (anti-VEGF) therapy, or insufficient post-SRT follow-up (<3 months). After applying the eligibility criteria, 32 eyes were included in the final analysis.

The mean BCVA significantly improved from 0.29 ± 0.30 logMAR at baseline to 0.20 ± 0.29 logMAR at 3 months post-SRT (*p* = 0.006). From baseline to 3 months, the mean CFT significantly decreased from 284.7 ± 91.3 to 165.7 ± 94.8 μm (*p* < 0.001). The mean SRF and PED heights decreased significantly from 150.5 ± 74.6 to 20.9 ± 48.3 μm (*p* < 0.001) and from 101.7 ± 96.9 to 33.3 ± 37.6 μm (*p* < 0.001). Additionally, the mean MD of retinal sensitivity showed a non-significant improvement from baseline to 3 months (*p* = 0.108). The reduction in SFCT at 3 months was not significant (*p* = 0.104) (Table 3).

**Figure 3:**
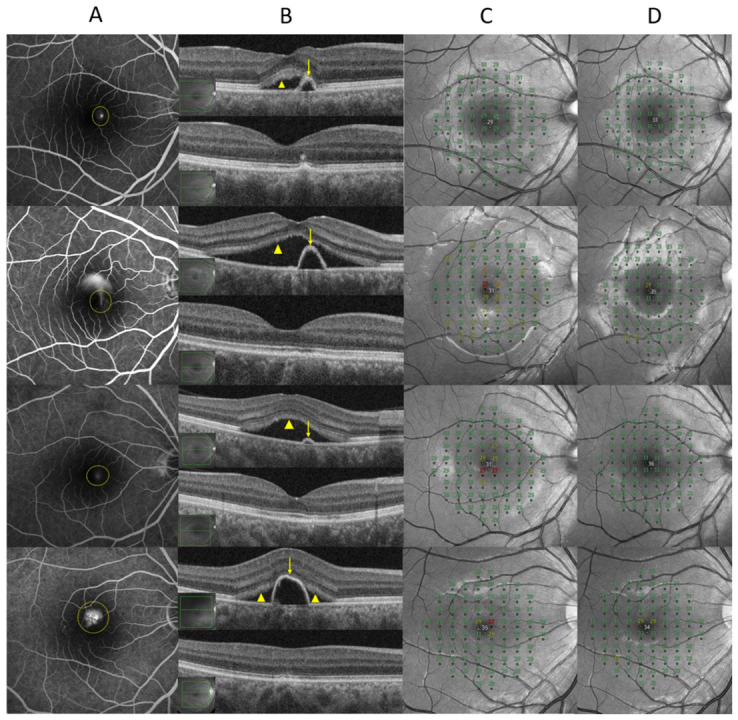
Representative multimodal images of four additional patients (Cases 2–5) with central serous chorioretinopathy demonstrating complete resolution of pigment epithelial detachment (PED) and subretinal fluid (SRF) following selective retina therapy (SRT). (**A**) Late-phase fluorescein angiography highlighting PED-associated leakage sites (yellow circles). (**B**) Baseline optical coherence tomography (OCT) images (**top**) showing serous PED (yellow arrows) with associated SRF (yellow arrowheads) and corresponding 3-month post-SRT OCT images (**bottom**) demonstrating complete flattening of the PED and resolution of SRF. (**C**) Baseline retinal sensitivity maps obtained by microperimetry. (**D**) Retinal sensitivity maps at 3 months after SRT demonstrating maintained or improved pointwise sensitivities (dB) within the treated areas. Numbers indicate retinal sensitivity values (dB). Red and dark-colored areas in the Compass microperimetry maps indicate relatively decreased retinal sensitivity. Each row (Cases 2–5) represents an individual patient.

**Figure 4:**
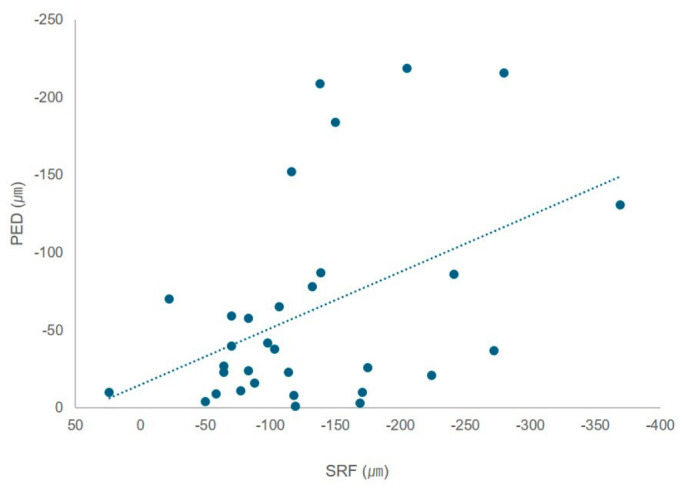
Scatter plot showing the correlation between changes in pigment epithelial detachment (PED) height and subretinal fluid (SRF) height from baseline to 3 months after selective retina therapy. Negative values indicate reductions in PED and SRF heights relative to baseline (Pearson r = 0.446, *p* = 0.011). Each point represents one eye.

Complete SRF resolution was observed in 12 (37.5%), 17 (53.1%), and 25 (78.1%) eyes at 1, 2, and 3 months post-SRT, respectively. PEDs were completely resolved in five (15.6%), nine (28.1%), and 12 (37.5%) eyes at 1, 2, and 3 months after SRT, respectively (Figure 3). The reductions in PED and SRF heights showed a moderate positive correlation (Pearson’s correlation coefficient, r = 0.446, *p* = 0.011) (Figure 4). Four eyes showed multiple leakage-associated PEDs, and in these cases, the PED with the greatest height was selected for analysis according to the predefined measurement protocol.

Subgroup analyses according to prior anti-VEGF treatment status demonstrated no significant differences in the main clinical outcomes at 3 months after SRT between eyes with and without previous anti-VEGF treatment (Table 4).

At 3 months, no test location within the SRT-treated area of any eye exhibited a loss in retinal sensitivity ≥8 dB relative to baseline. Re-treatment was performed in 10 of the 32 eyes (31.3%) at 2 months post-SRT. Among the twelve eyes that achieved complete SRF resolution at 1 month, recurrence was observed in two eyes at the 2-month visit. Both eyes underwent re-treatment at 2 months, and complete SRF resolution was again achieved at 3 months.

In the first SRT, the mean pulse energy for treatment spots was 155.3 ± 30.2 μJ (range: 100–200 μJ). The mean number of treatment spots of the first SRT was 27.8 ± 22.5. All treatment spots were invisible during irradiation. When the micropulse count was set to three at the same pulse energy, the resulting test spots appeared less visible or even invisible compared with the barely visible test spots produced by 10 micropulses, suggesting a tendency toward a weaker fluorescein angiographic response (Figure 1).

On FAF, barely visible SRT test spots exhibited either no change or mild hypoautofluorescence at 1 h post-irradiation. By 1 month, they appeared hyperautofluorescent, which progressively attenuated over the 3-month follow-up. Evaluation of macular treatment spots was partly limited by overlying SRF. Nevertheless, no areas of pronounced hypoautofluorescence suggestive of retinal pigment epithelium atrophy were detected during follow-up (Figure 1). No SRT-related adverse events, including retinal burns or hemorrhage, were observed throughout the study period.

## 4. Discussion

We included only eyes with CSC accompanied by serous PEDs that demonstrated fluorescein leakage in this study to evaluate the direct effects of SRT on PEDs. To our knowledge, this is an issue that has not been examined to date. At 3 months post-SRT, complete SRF resolution was achieved in 78.1% of eyes. The mean BCVA improved, and the mean CFT, SRF height, and PED height decreased significantly at 3 months. Although the mean MD of retinal sensitivity improved, it was not significant. Taken together, these findings suggest that SRT was associated with reductions in SRF and PED heights without evidence of short-term functional deterioration. However, given the absence of a control group, the potential influence of the natural course of CSC should also be considered when interpreting these findings.

In previous studies, SRT has demonstrated complete SRF resolution rates ranging from 59% to 80.8% at 3 months in patients with CSC [15,17,20,21,22,23]. The SRF resolution rate observed in the present cohort of CSC eyes with serous PEDs was comparable to these previously reported outcomes, despite differences in study design and patient selection. Since longstanding SRF in CSC can lead to irreversible vision loss through photoreceptor degeneration and RPE atrophy, the primary therapeutic goal has traditionally focused on complete SRF resolution. However, beyond SRF itself, prolonged RPE detachment from the underlying choroid may independently precipitate RPE dysfunction and subsequent atrophic changes [11]. PEDs are closely associated with pachychoroid features, including dilated Haller vessels, and may occur with or without concurrent SRF [7,11].

Importantly, the clinical significance of PEDs extends beyond their potential for spontaneous resolution. In a 49-month natural history study of CSC-related PEDs, although 65% of PEDs resolved without intervention, RPE atrophy developed in 86% of cases even after complete PED resolution, and subfoveal PEDs were associated with poorer visual prognosis [11,24]. These findings suggest that spontaneous PED resolution does not necessarily indicate structural or functional recovery of the RPE, underscoring the potential value of therapeutic strategies aimed at timely restoration of RPE–choroid apposition rather than observation alone.

SRT was directed to the PED-associated leakage in our retrospective cohort. Over 3 months of follow-up, no treatment-related structural complications, specifically RPE atrophy or tears, were detected on OCT or FAF, including at sites where PEDs achieved resolution. Although the observation period was limited, these findings suggest that short-term PED resolution post-SRT can be achieved without evidence of early RPE atrophy at the treated loci. A longer follow-up is required to determine whether SRT modifies the high risk of late RPE atrophy reported after spontaneous PED resolution in longstanding disease.

To date, only a limited number of studies have described morphological changes in PEDs following laser-based treatments for CSC. In a retrospective series, Hwang et al. reported complete resolution of subfoveal PEDs in 28 of 35 eyes (80%) at 1 month after half-dose PDT [25]. In the PLACE trial, which compared half-dose PDT with high-density SML in chronic CSC, complete resolution of the highest macular PED was observed in 36.5% of patients treated with half-dose PDT and in 13.0% of those treated with high-density SML at 7–8 months after the first treatment [26].

These findings provide a reference for the range and timing of PED changes reported after established laser-based therapies. In our cohort, complete PED resolution was observed in 37.5% of eyes at 3 months after SRT. Given the absence of a control group and the variability in the natural course of CSC-related PEDs, these findings should be interpreted descriptively.

Regarding quantitative changes in PED height, the PLACE trial reported median reductions in the highest PED height in both treatment groups. In the half-dose PDT group, reductions were 6.50 µm at the first evaluation visit (6–8 weeks) and 12.00 µm at the final evaluation visit (7–8 months). In the high-density SML group, corresponding reductions were 1.00 µm and 3.00 µm, respectively. These findings provide descriptive context for the range of PED changes reported after laser-based therapies in chronic CSC.

In the present study, mean decreases in PED height of 52.8 µm and 68.4 µm were observed at 2 and 3 months after SRT, respectively. Because of differences in study design, baseline PED characteristics, inclusion criteria, and follow-up intervals, direct quantitative comparisons across studies are not appropriate. Accordingly, the observed PED height changes following SRT should be interpreted descriptively within the context of this selected cohort rather than as evidence of relative efficacy compared with currently used treatment modalities.

While the exact mechanism by which PDT affects PED in CSC remains unclear, its primary effect appears to be related to choriocapillaris damage and hypoperfusion caused by free-radical formation following intravenous administration of the photosensitizer verteporfin. Long-term remodeling of choroidal vessels underlying the damaged RPE may subsequently affect PEDs [11,27]. Moreover, the mechanism of action of SML remains uncertain. The laser energy of the SML is believed to stimulate the secretion of heat shock proteins within the RPE, which contributes to SRF removal [28]. Accordingly, SML may affect RPE function without causing thermal damage to the RPE.

To date, no multimodal imaging technique can detect laser lesions post-SML. However, SRT lesions can be clearly observed as hyperfluorescence on FFA because of structural RPE damage. Since the SRT-damaged area can subsequently be covered by the migration and proliferation of neighboring healthy RPE cells, this process may contribute to sealing the leakage point by restoring the outer blood–retinal barrier [14]. SRT may induce structural changes through direct RPE damage and subsequent repair processes, which are mechanistically distinct from those proposed for PDT or SML.

In our study, reductions in PED and SRF height were moderately and positively correlated, suggesting a potential association between structural changes following SRT and SRF resolution. Although the correlation was modest, a decrease in PED height may reflect changes associated with reduced leakage activity and subsequent SRF absorption after SRT.

Notably, to our knowledge, the only previous report on CSC with serous PED treated with SRT included five eyes and documented SRF resolution in four eyes at 1 month. However, quantitative PED height metrics were not provided, and no meaningful PED flattening was observed [29]. Differences between that report and the present findings are difficult to interpret. Still, they may reflect the limited generalizability of the previous small case series, as well as differences in case selection and treatment parameters.

Beyond these anatomical considerations, the functional implications of SRT are also clinically relevant. Mean BCVA improved following SRT, whereas the mean MD of retinal sensitivity showed a numerical increase without statistical significance. In addition, no pointwise decrease of 8 dB or greater in retinal sensitivity was detected within the SRT-treated area, which is consistent with the absence of detectable SRT-related photoreceptor damage. Taken together, these findings suggest that SRT was not associated with measurable short-term functional deterioration. Additionally, as reported previously [15,17], mean SFCT did not change significantly after SRT, supporting the concept that SRT primarily exerts its effects at the level of the RPE rather than the choroid.

Our study had several limitations. First, the retrospective design, absence of a control group, and relatively short follow-up period of 3 months limit the ability to assess the long-term durability of PED resolution, recurrence, and potential late complications such as RPE atrophy or choroidal neovascularization. These factors also make it difficult to distinguish the treatment effect of SRT from the natural course of CSC, which may include spontaneous fluctuation or partial resolution of SRF. Therefore, the long-term clinical implications of the observed anatomical improvements remain uncertain.

Second, the sample size was relatively small, which may limit the statistical power, generalizability, and reliability of the findings. A subset of eyes overlapped with a previously reported prospective cohort; however, the present study addressed a distinct research question focusing on PED-associated leakage and PED-specific morphological outcomes after SRT. Third, the maximum baseline PED height in the present cohort was 315 µm, and the treatment response of CSC eyes with larger PEDs remains uncertain. Fourth, a considerable proportion of eyes (43.8%) had a history of prior anti-VEGF treatment, reflecting real-world clinical practice. Although subgroup analyses showed no significant differences in 3-month clinical outcomes according to prior anti-VEGF treatment status, residual confounding effects related to previous treatment exposure cannot be completely excluded.

Despite these limitations, our findings suggest that SRT was associated with early reduction or resolution of serous PEDs in CSC. Because some CSC-associated serous PEDs may become vascularized over time [30], anatomical resolution of PEDs may be clinically relevant in the overall disease course. However, the present data do not allow any inference regarding the long-term effects of SRT, including the risk of PED vascularization.

## 5. Conclusions

In summary, SRT was associated with a reduction in serous PED height in patients with CSC, and PED reduction was correlated with SRF resolution. These findings should be interpreted cautiously given the absence of a control group and the potential for spontaneous changes in SRF and PED. Further prospective studies with larger cohorts and longer follow-up are needed to confirm the durability and clinical significance of these findings.

## Figures and Tables

**Table 1:** Inclusion and exclusion criteria.

Inclusion Criteria
Visual symptoms for ≥3 months Presence of fovea-involving subretinal fluid on OCT
Presence of serous PED showing homogeneous hyporeflective signal on OCT Angiographic leakage primarily originating from serous PED
Availability of ≥3 months of post-SRT follow-up
Exclusion Criteria
Presence of other macular diseases, including non-CSC entities such as AMD, PCV, and pathological myopia
Any evidence of choroidal neovascularization on multimodal imaging (OCT, FFA, ICGA, or OCT angiography) Fibrous or vascular PEDs showing heterogeneous hyperreflective signals suggestive of neovascular tissue on OCT
Serous PEDs with angiographic leakage not primarily originating from PED
History of conventional laser photocoagulation or PDT in macula area
History of intravitreal anti-VEGF injection within 3 months before SRT
History of local or systemic steroids, acetazolamide, or aldosterone antagonists within 12 months before SRT
History of intraocular surgery within 12 months before SRT

OCT, optical coherence tomography; PED, pigment epithelial detachment; SRT, selective retina therapy; AMD, age-related macular degeneration; PCV, polypoidal choroidal vasculopathy; FFA, fundus fluorescein angiography; ICGA, indocyanine green angiography; PDT, photodynamic therapy; VEGF, vascular endothelial growth factor.

**Table 2:** Baseline demographics and clinical characteristics of 32 eyes from 32 patients with central serous chorioretinopathy accompanied by serous pigment epithelial detachment.

Patient Characteristics	Values
Number of patients (eyes), *n*	32 (32)
Age (years), mean ± SD	45.9 ± 10.9
Sex, *n* (%)	
Male	25 (78.1%)
Female	7 (21.9%)
Symptom duration in months, mean ± SD	9.3 ± 11.3
Previous treatments	
Prior intravitreal anti-VEGF injection (≥3 months before SRT), *n* (%)	14 (43.8%)
Prior spironolactone (≥12 months before SRT), *n* (%)	1 (3.1%)
Shape of pigment epithelial detachment	
Dome, *n* (%)	27 (84.4%)
Flat, *n* (%)	5 (15.6%)
BCVA (logMAR), mean ± SD	0.29 ± 0.30
Central foveal thickness (µm), mean ± SD	284.7 ± 91.3
Subfoveal choroidal thickness (µm), mean ± SD	425.5 ± 99.0
Subretinal fluid height (µm), mean ± SD	150.5 ± 74.6
Pigment epithelial detachment height (µm), mean ± SD	101.7 ± 96.9
MD (dB) of microperimetry, mean ± SD	−1.62 ± 2.33

BCVA, best-corrected visual acuity; SD, standard deviation; VEGF, vascular endothelial growth factor; MD, mean deviation; logMAR, logarithm of the minimum angle of resolution.

**Table 3:** Changes in ocular parameters over time.

	Baseline	1 Month	2 Months	3 Months
BCVA (logMAR)				
Mean ± SD	0.29 ± 0.30	0.26 ± 0.32	0.23 ± 0.32	0.20 ± 0.29
*p*-value		0.154	0.012 *	0.006 *
CFT (μm)				
Mean ± SD	284.7 ± 91.3	202.0 ± 96.3	182.0 ± 84.5	165.7 ± 94.8
*p*-value		<0.001 *	<0.001 *	<0.001 *
SFCT (μm)				
Mean ± SD	425.5 ± 99.0	418.1 ± 99.2	416.7 ± 99.1	414.3 ± 98.9
*p*-value		0.319	0.178	0.104
SRF height (μm)				
Mean ± SD	150.5 ± 74.6	80.1 ± 97.8	53.0 ± 69.3	20.9 ± 48.3
*p*-value		0.001 *	<0.001 *	<0.001 *
PED height (μm)				
Mean ± SD	101.7 ± 96.9	62.2 ± 60.1	48.9 ± 54.3	33.3 ± 37.6
*p*-value		0.010 *	0.003 *	<0.001 *
MD (dB) of retinal sensitivity				
Mean ± SD	−1.62 ± 2.33			−0.97 ± 3.25
*p*-value				0.108

BCVA, best-corrected visual acuity; CFT, central foveal thickness; SFCT, subfoveal choroidal thickness; SRF, subretinal fluid; PED, pigment epithelial detachment; MD, mean deviation; SD, standard deviation; logMAR, logarithm of the minimum angle of resolution. * *p* < 0.05.

**Table 4:** Comparison of changes in 3-month clinical outcomes after selective retina therapy according to prior anti-VEGF treatment status.

	Prior Anti-VEGF Treatment, *n* = 14	No Prior Anti-VEGF Treatment, *n* = 18	*p*-Value *
BCVA (logMAR)			
Mean ± SD	−0.09 ± 0.22	−0.10 ± 0.17	0.577
CFT (μm)			
Mean ± SD	−85.0 ± 105.57	−148.9 ± 115.9	0.053
SRF height (μm)			
Mean ± SD	−102.4 ± 65.2	−150.6 ± 88.7	0.106
PED height (μm)			
Mean ± SD	−58.9 ± 106.3	−76.1 ± 72.0	0.224
Complete SRF resolution			
*n* (%)	9 (64.3%)	16 (88.9%)	0.195
Complete PED resolution			
*n* (%)	3 (21.4%)	9 (50.0%)	0.167

VEGF, vascular endothelial growth factor; BCVA, best-corrected visual acuity; CFT, central foveal thickness; SRF, subretinal fluid; PED, pigment epithelial detachment. Continuous variables were compared using Mann–Whitney U test, and categorical variables were compared using Fisher’s exact test. * *p* < 0.05.

## Data Availability

The datasets supporting this article are subject to privacy and ethical restrictions, and therefore cannot be made publicly available. Requests for clarification may be directed to the corresponding author.

## Supplementary Materials

The following supporting information can be downloaded at: https://www.mdpi.com/article/10.3390/jcm15103905/s1, Table S1: Sensitivity Analysis of Clinical Outcomes after Selective Retina Therapy Excluding the Nine Eyes Overlapping with the Previously Published Cohort.

## Abbreviations

The following abbreviations are used in this manuscript:

SRT	Selective retina therapy
CSC	Central serous chorioretinopathy
PED	Pigment epithelial detachment
BCVA	Best-corrected visual acuity
CFT	Central foveal thickness
SRF	Subretinal fluid
RPE	Retinal pigment epithelium
OCT	Optical coherence tomography
PDT	Photodynamic therapy
VEGF	Vascular endothelial growth factor
SML	Subthreshold micropulse laser
FFA	Fundus fluorescein angiography
ICGA	Indocyanine green angiography
