# Supplementary material for: Effects of Selective Retina Therapy on Central Serous Chorioretinopathy with Serous Pigment Epithelial Detachments
Source: J Clin Med. 2026 May 19;15(10):3905. doi: 10.3390/jcm15103905 (PMC13207491; doi:10.3390/jcm15103905)
Supplement: Supplementary file 1 [file jcm-15-03905-s001.zip › jcm-4286594-supplementary.pdf]

Supplementary Table S1. Sensitivity analysis of clinical outcomes after selective retina therapy excluding the nine eyes overlapping with the previously published cohort.

	Baseline	1 month	2 months	3 months
BCVA (logMAR)				
Mean ± SD	0.31 ± 0.33	0.27 ± 0.35	0.23 ± 0.33	0.21 ± 0.29
p-value		0.218	0.021*	0.018*
CFT (μm)				
Mean ± SD	273.0 ± 88.9	198.7 ± 93.0	174.6 ± 80.4	150.6 ± 64.5
p-value		0.002*	< 0.001*	< 0.001*
SFCT (μm)				
Mean ± SD	395.7 ± 95.4	394.3 ± 100.3	397.1 ± 104.8	391.9 ± 102.4
p-value		0.828	0.784	0.449
SRF height (μm)				
Mean ± SD	142.0 ± 72.2	72.2 ± 78.8	46.4 ± 62.3	15.6 ± 33.3
p-value		< 0.001*	< 0.001*	< 0.001*
PED height (μm)				
Mean ± SD	103.0 ± 105.3	64.9 ± 59.8	51.5 ± 54.8	37.0 ± 41.2
p-value		0.048*	0.016*	0.002*
MD (dB) of retinal sensitivity				
Mean ± SD	-1.58 ± 2.48			-1.33 ± 3.79
p-value				0.516

BCVA, best-corrected visual acuity; CFT, central foveal thickness; SFCT, subfoveal choroidal thickness; SRF, subretinal fluid; PED, pigment epithelial detachment; MD, mean deviation; SD, standard deviation; logMAR, logarithm of the minimum angle of resolution. *p < 0.05
